# Copper/Zinc Superoxide Dismutase from the Crocodile Icefish *Chionodraco hamatus*: Antioxidant Defense at Constant Sub-Zero Temperature

**DOI:** 10.3390/antiox9040325

**Published:** 2020-04-17

**Authors:** Evangelia Chatzidimitriou, Paola Bisaccia, Francesca Corrà, Marco Bonato, Paola Irato, Laura Manuto, Stefano Toppo, Rigers Bakiu, Gianfranco Santovito

**Affiliations:** 1 Institute of Natural Resource Sciences, ZHAW Zurich University of Applied Sciences, 8820 Wädenswil, Switzerland; evangelia.chatzidimitriou@zhaw.ch; 2 Department of Biology, University of Padova, 35131 Padova, Italy; paola.bisaccia@studenti.unipd.it (P.B.); francesca.corra@unipd.it (F.C.); marco.bonato@unipd.it (M.B.); paola.irato@unipd.it (P.I.); 3 Department of Molecular Medicine, University of Padova, 35131 Padova, Italy; laura.manuto@studenti.unipd.it (L.M.); stefano.toppo@unipd.it (S.T.); 4 CRIBI Biotech Centre, University of Padova, 35131 Padova, Italy; 5 Department of Aquaculture and Fisheries, Agricultural University of Tirana, 1000 Tiranë, Albania; bakiurigers@gmail.com

**Keywords:** Antarctica, *Chionodraco hamatus*, cold adaptation, gene expression, molecular evolution, protein purification, superoxide dismutase, teleosts

## Abstract

In the present study, we describe the purification and molecular characterization of Cu,Zn superoxide dismutase (SOD) from *Chionodraco hamatus*, an Antarctic teleost widely distributed in many areas of the Ross Sea that plays a pivotal role in the Antarctic food chain. The primary sequence was obtained using biochemical and molecular biology approaches and compared with Cu,Zn SODs from other organisms. Multiple sequence alignment using the amino acid sequence revealed that Cu,Zn SOD showed considerable sequence similarity with its orthologues from various vertebrate species, but also some specific substitutions directly linked to cold adaptation. Phylogenetic analyses presented the monophyletic status of Antartic Teleostei among the Perciformes, confirming the erratic differentiation of these proteins and concurring with the theory of the “unclock-like” behavior of Cu,Zn SOD evolution. Expression of *C. hamatus* Cu,Zn SOD at both the mRNA and protein levels were analyzed in various tissues, highlighting the regulation of gene expression related to environmental stress conditions and also animal physiology. The data presented are the first on the antioxidant enzymes of a fish belonging to the Channichthyidae family and represent an important starting point in understanding the antioxidant systems of these organisms that are subject to constant risk of oxidative stress.

## 1. Introduction

Various proteins and compounds defend cells from reactive oxygen species (ROS). Superoxide dismutases (SODs, EC 1.15.1.1) are metalloenzymes with the main physiological role of preventing oxidative stress by catalyzing the partitioning of the superoxide anion (^•^O_2_^−^) into hydrogen peroxide and molecular oxygen [1,2].

In animals, there are three known types of SODs: manganese SOD (Mn SOD or SOD2), which is localized in the mitochondrial matrix, and two Cu,Zn SODs, namely intracellular SOD (IC SOD or SOD1) and extracellular SOD (EC SOD or SOD3) with respect to their cellular localization [3,4].

Cu,Zn SODs show extremely conserved structural and functional parameters, such as their kinetics and their electrostatic potential surrounding the active site, a feature that modulates the catalytic mechanism of these enzymes [5,6]. On the contrary, SODs were reported to exhibit exclusive properties in fish, such as a variability in isoelectric point (pI) values over a wide pH range [7,8].

Although SOD1s from many fish have been characterized [9,10,11], very limited knowledge exists regarding this protein in Antarctic fish [12] and nothing is known of those belonging to the Channichthyidae family, the icefish. In this work, we describe the molecular characterization of SOD1 from *Chionodraco hamatus*, a teleost living in the cold waters surrounding the Antarctic continental shelf. We compared the obtained sequence with orthologs from other fish, with the aim of evaluating differences related to different evolutionary histories. We also employed in silico approaches to investigate Antarctic fish SOD1s for the possible presence of molecular cold adaptation characteristics.

In this regard, SODs are interesting because they are considered ancient enzymes due to their unique evolutionary history [13,14]. In particular, Cu,Zn SOD rapidly evolved in relatively recent times, in contrast to Mn and Fe SODs that seem to have evolved at a relatively constant rate throughout the entire history of eukaryotes [14].

A comparative study on the evolutionary aspects of Cu,Zn SOD amino acid sequences, including bacteria and mammals, revealed that this protein contains both invariant and variable regions [15]. The former are directly involved in metal ligand binding or are responsible for maintaining the structure and function of the active site, dimer contacts, and β-barrel folds. The presence of numerous invariant residues, even in alignments from very distant species, make this enzyme one of the most evolutionary stable globular proteins characterized to date [16]. Nonetheless, the variable regions permit the evaluation of divergences between various groups of the animal kingdom.

Finally, we characterized basal mRNA and active protein levels in various organs and tissues to obtain some new insight into the physiological role of SOD1 in *C. hamatus*.

## 2. Materials and Methods

### 2.1. Ethical Procedures

The sample collection and animal research conducted in this study comply with Italian Ministry of Education, University and Research regulations concerning activities and environmental protection in Antarctica and with the Protocol on Environmental Protection to the Antarctic Treaty, Annex II, Art. 3. All experiments have been performed in accordance with the U.K. Animals (Scientific Procedures) Act, 1986 and associated guidelines, EU Directive 2010/63/EU and Italian DL 2014/26 for animal experiments.

### 2.2. Experimental Animals

Adult samples of *C. hamatus* were collected in the proximity of Mario Zucchelli Station in Terra Nova Bay, Antarctica (74°42′ S, 167°7′ E) and kept in aquaria supplied with aerated seawater at approximately 0 °C. After a destress period of seven days, ten specimens were euthanized (tricainemethanesulfonate, MS-222; 0.2 g L^−1^) and samples of gills, heart, liver, skeletal muscle and spleen tissues were excised, quickly frozen in liquid nitrogen and stored at −80 °C.

All the activities on animals performed during the Italian Antarctic Expedition are under the control of a PNRA Ethics Referent, which acts on behalf of the Italian Ministry of Foreign Affairs. In particular, the required data are the following. Project identification code: PNRA16_00099. Name of the ethics committee or institutional review board: Italian Ministry of Foreign Affairs. Name of PNRA Ethics Referent: Dr. Carla Ubaldi, ENEA Antarctica, Technical Unit (UTA). Date of approval: 12/07/2017.

### 2.3. Primers Design, RNA Extraction, cDNA Synthesis, RACE, Cloning, and Sequencing of SOD1 mRNA

Amino acid and nucleotide sequences of SOD1 from notothenioid fish obtained from the NCBI database were aligned by MUSCLE to identify conserved domains for primer design [17]. The species considered were *Cottoperca gobio* (channel bull blenny), *Notothenia coriiceps* (rockcod), and *Trematomus bernacchii* (emerald rockcod). The primer sequences, shown in Table S1, were analyzed using IDT Oligo analyzer (Coralville, IA, USA) (http://eu.idtdna.com/analyzer/Applications/OligoAnalyzer/).

Total RNA was purified from various tissues of *C. hamatus* using TRIzol^®^ reagent (Invitrogen, Thermo Fisher Scientific, Waltham, MA, USA) according to the manufacturer’s protocol [18]. Further purification was performed with 8 M LiCl in order to remove glucidic contaminants. RNA concentration and integrity were assessed using a Nanodrop ND-1000 spectrophotometer (Thermo Fisher Scientific) and an Agilent Bioanalyzer 2100 (Agilent, Santa Clara, CA, USA) alongside an RNA 6000 Nano, respectively. The first strand of cDNA was reverse-transcribed from 1 µg total RNA at 42 °C for 1 h in a 20 μL reaction mix containing 1 μL of ImProm-IITM Reverse Transcriptase (Promega, Madison, WI, USA) and 0.5 μg oligodT anchor primer. Various forward and reverse primers, listed in Table S1, were applied to 50 ng of cDNA in PCR reactions. The PCR program was as follows: 95 °C for 2 min, 35 cycles of 95 °C for 30 s, 55 °C for 30 s, and 72 °C for 1 min, and 72 °C for 10 min.

cDNA synthesis for 3′ rapid amplification of cDNA ends (RACE) analysis was primed with Oligo-dT adaptor primer (Table S1). PCR amplification was performed using various nested sense and anchoring primers (Table S1) according to the following thermal program: 94 °C for 2 min, then 36 cycles of 94 °C for 30 s, 62 °C for 30 s, and 72 °C for 90 s.

The 5′ end of the cDNA was amplified using the 5′RACE System kit (Invitrogen) following the manufacturer’s instructions. The cDNA was synthesized using the primer 5′RACE RE1 (Table S1) and amplified in two PCR steps. The first step, using the anti-sense primer 5′RACE RE1 and anchor primer Abridged Anchor Primer (AAP), was performed according to the following program: 94 °C for 2 min, 35 cycles of 94 °C for 1 min, 56 °C for 1 min, and 72 °C for 2 min, then 72 °C for 7 min. One microliter of the obtained amplicon was applied to a nested amplification using the primers 5′RACE RE2 and Abridged Universal Amplification Primer (AUAP) (Table S1). The PCR program was the same as the previous amplification step.

All PCR products were purified with the NucleoSpin Extract 2 in 1 (Macherey-Nagel, Düren, Germany), ligated into the pGEM^®^-T Easy Vector (Promega) and cloned in XL1-Blue *Escherichia coli* (Invitrogen). DNA from positive clones were sequenced at BMR Genomics (Padova, Italy) on an ABI PRISM 3700 DNA Analyzer (Applied Biosystems, Foster City, CA, USA). 

### 2.4. SOD Activity Determination

SOD activity was measured by the Beauchamp and Fridovich method [19]. One unit of SOD was defined as the amount of enzyme required for 50% inhibition of nitroblue tetrazolium conversion. The activity was normalized to the total protein concentration and measured by the Lowry method [20].

### 2.5. Protein Purification

A pool of livers was washed in cold physiological solution and homogenized with a Polytron (Kinematica, Luzern, Switzerland) in 0.1 M Tris-HCl, pH 7.5, containing 0.2 mM phenyl-methylsulfonyl fluoride (5 volumes/g). The homogenate was centrifuged in two steps, with the first at 48,000× *g* at 4 °C for 60 min and the obtained supernatant centrifuged at 27,000× *g* at 4 °C for 30 min. The new supernatant was dialyzed in 20 mM Tris-HCl, pH 7.8, and filtered by particle size of 0.22 µm.

The sample was loaded on a gel filtration chromatography system with a Sephadex G-75 column (26 × 850 mm; Merck, Darmstadt, Germany), then equilibrated and eluted with 20 mM Tris-HCl buffer, pH 7.8. SOD activity was measured in all fractions, and the positive ones were pooled and dialyzed in 5 mM Tris-HCl buffer, pH 7.8. Further separation was performed on a DEAE Sephacel A-50 anion exchange column (20 × 150 mm; Merck) and equilibrated with the same buffer, eluting the sample in 150 mL of a 0–200 mM NaCl linear gradient.

Fractions with activity greater than 50 units of SOD per mg total protein were pooled and equilibrated with 5 mM Tris-HCl, pH 7.5, and then loaded in an fast protein liquid chromatography (FPLC) system (Thermo Fisher Scientific) with a Mono Q HR 5/5 anion exchange column and equilibrated with the same buffer, eluting the sample in a 0-400 mM NaCl linear gradient (pH 7.5). After equilibration of the samples at pH 7.8, this chromatographic step was repeated, eluting the column in a 0–200 mM NaCl linear gradient (Figure S1). Fractions with SOD activity were pooled and used for physiochemical analysis. 

### 2.6. Determination of Molecular Properties

The molecular mass of native protein was determined by gel exclusion chromatography on a Sephadex G-75 column. Bovine serum albumin (MW 67,000), ovalbumin (MW 43,000), chymotrypsinogen A (MW 25,000), and cytochrome c (MW 12,300) were used as molecular weight standards (Merck).

The protein was separated by electrophoresis in 7.5% polyacrylamide gels, as previously described [21]. Gels were stained for protein with Coomassie brilliant blue [22] and for SOD activity according to the Beauchamp and Fridovich method [19] 

The molecular weight of a single monomer was estimated by SDS/PAGE using the PhastGel gradient 10–15 in 1% SDS and 0.1 M β-mercaptoethanol [23], which stained the gel silver. Bovine phosphorylase (MW 94,000), serum albumin (MW 67,000), ovalbumin (MW 43,000), carbonic anhydrase (MW 30,000), trypsin inhibitor (MW 20,100), and alpha-lactoalbumin (MW 14,000) were used as molecular weight standards (Merck). The pI was determined by isoelectric focusing on a pH 3–9 gradient gel, as previously described [24]. The presence of copper and zinc linked to the protein was verified by measuring the concentration of these metals by atomic absorption spectroscopy using a Perkin Elmer 5100 graphite furnace atomic absorption spectrometer.

Mass spectrum of the purified protein was obtained by matrix-assisted laser desorption ionization time of flight (MALDI-TOF) with a Bruker Reflex-III spectrometer, dissolving the sample in 0.1% trifluoroacetic acid. Bovine serum albumin (MW 67,000) and trypsinogen (MW 24,000) were used as molecular weight standards (Merk). All these proteins are from bovine and were purchased from Merck, Darmstadt, Germany.

### 2.7. Protein Sequence Analyses

Sample purity was assessed before sequence analysis by capillary electrophoresis under both acidic (0.1 M sodium phosphate, pH 2.5) and alkaline (0.1 M sodium phosphate, pH 8.0) conditions. The N-terminal amino acid sequence of the protein was identified by automated Edman degradation on an Applied Biosystems 477A sequencer equipped with an online phenylthiohydantoin amino acid analyzer, using polybrene as a carrier for the protein sample. Sequence analysis was conducted using the dedicated software tool (Applied Biosystems).

The purified protein was concentrated by drying in a SpeedVac (Thermo Fisher Scientific) and alternatively digested with trypsin, thermolysin, or V8 protease. Dried samples were re-suspended in 50 mM ammonium bicarbonate for tryptic digestion only.

### 2.8. Phylogenetic Analyses

Both amino acid and nucleotide sequences of SOD1s from *C. hamatus* were used for phylogenetic analyses, together with SOD1 sequences from other species available in the GenBank database (Table S2).

Multiple alignment of SOD1 sequences was obtained using the T-Coffee multiple sequence alignment package (Comparative Bioinformatics Group, Barcelona, Spain) [25]. Although this method is based on the widely used progressive approach to multiple alignment, it was chosen because it is significantly more accurate than the most common alternatives, with a negligible sacrifice of speed [25].

Statistical selection of best-fit models of nucleotide substitution were carried out using the jModelTest 2 [26]. Three types of information criteria (Akaike Information Criterion - AIC, Corrected Akaike Information Criterion (cAIC) and Bayesian Information Criterion (BIC)) and 88 candidate models were used for this analysis. The best-fit model of analyzed protein evolution was selected according to ProtTest 3 [27]. In these statistical analyses, the three previously mentioned criteria and 122 candidate models were used.

Phylogenetic trees were built using the Bayesian inference (BI) method applied in Bayes 3.2 [28] and the maximum likelihood (ML) method applied in PhyML 3.0 [29]. For the BI method, four independent runs, each with four simultaneous Markov Chain Monte Carlo (MCMC) chains, were performed for 1,000,000 generations sampled every 1000 generations. For the ML method, bootstrap analyses were performed on 100,000 trees using both kinds of tree topology improvement, i.e., nearest neighbor interchange (NNI) and subtree pruning and regrafting (SPR). The annotated phylogenetic trees were displayed using FigTree v1.3 software (GitHub, San Francisco, CA, USA).

Finally, we employed the mechanistic empirical model (MEC) [30] to consider the different probabilities of amino acid replacement based on the Whelan and Goldman empirical replacement matrix (WAG), while also estimating the codon rate matrix, thus allowing the positions undergoing radical amino acid exchanges to acquire higher dN rates than those with less radical exchanges.

The Selecton-3D web server (http://selecton.tau.ac.il) was used in order to map the codonwise × estimates against human protein tertiary structure (Protein Data Bank: 5yto).

Tests of equality of evolutionary implemented in the HyPhy software package (Temple University Commonwealth, Philadelphia, PA, USA) [31] were performed for molecular clock analyses. 

### 2.9. Molecular Modeling

Structural models of SOD1 isoforms from *C. hamatus*, *T. bernacchii*, *N. coriiceps,* and *Stegastes partitus* were obtained. For each of the analyzed proteins, the SWISS-MODEL [32] was used to build a structural model starting from a manual selected template based on the best QMEAN score, manual refinement of the alignment between the target and the template, and X-ray resolution. For each protein, it was possible to select and use the same template, thereby simplifying the evaluation and comparison of the electrostatic surface potential of the built models. As shown in Table S3, all selected templates presented a sequence identity higher than 60% and a QMEAN greater than –4.

Electrostatic surface potential was calculated for the energy minimized models using the programs Protein Data Bank To Per-atom charge (Q) and radius (R) format (PDB2PQR) [33] and Adaptive Poisson-Boltzmann Solver (APBS) [34] according to the nonlinear Poisson–Boltzmann equation and contoured at ±2 kT/e. The predicted model enriched with electrostatic and solvation properties was analyzed on Visual Molecular Dynamics (VMD) [35] and a comparison among similar proteins of different species was performed. The highlighted differences were further investigated through a multiple alignment analysis performed using Clustal Omega (EMBL-EBI, Hinxton, UK) [36].

### 2.10. Semiquantitative RT-PCR Analysis

SOD1 mRNA expression was evaluated by performing semiquantitative RT-PCR analysis, using *C. hamatus* β-actin (GenBank accession number: AJ532569.1) as the housekeeping gene. cDNAs for both genes were amplified with the specific primers reported in Table S1. The following thermal program was applied for the PCR amplifications: 95 °C for 2 min, a variable number of cycles (42 for SOD1, 40 for β-actin) of 95 °C for 30 s, 30 s at a specific melting temperature, and 72 °C for 1 min. For both genes, the number of amplification cycles was optimized to make sure that amplicons were quantified during the exponential phase.

The amplicons were separated by 1.5% GelRed-stained (Biotium, Fremont, CA, USA) agarose gel electrophoresis and the relative intensities of each band were quantified with the densitometric software Quantity-one using a quantitative ladder (Gene Ruler^TM^, Fermentas, Thermo Fisher Scientific). Transcript levels were reported (in arbitrary units, a.u.) as the ratio between SOD1 and β-actin expression in the same sample.

### 2.11. Statistical Analyses

Statistical analyses were performed with the PRIMER statistical program (PRIMER-e, Auckland, New Zealand). One-way ANOVA was followed by the Student–Newman–Keuls test to assess significant differences (*p* < 0.05). The data were expressed as the average of five analyzed specimens ± standard deviation (SD). 

## 3. Results

### 3.1. Organization of the C. hamatus Sod1 Gene

The complete cDNA sequence of the *C. hamatus* SOD1 gene (GenBank accession number AY736281.1) was obtained.

The primary transcript was 668 nt long. The 5′- and 3′- untranslated (UTR) regions consisted of 71 nt and 114 nt, respectively. The open reading frame included 483 nt and encoded a protein of 160 aa, with a deduced molecular weight of 16.68 kDa (Figure S2). The 3′-UTR region included a putative polyadenylation signal (AATAAA) at 649–654 nt. 

The amino acid sequence of SOD1 of *C. hamatus* showed most similar identities with the SOD1s of the Antarctic fish *N. coriiceps* and *T. bernacchii* (98.70% and 96.03%, respectively; Table S4).

### 3.2. Molecular Characterization of SOD1 Protein

From 22.5 g of liver tissue we obtained about 1.6 mg of SOD1, with a 90-fold increase in specific activity (Table S5). Polyacrylamide gel electrophoresis results demonstrated the purity of the purified protein.

Isoelectric focusing analysis showed the presence of a single protein fraction with an Ip value of pH 5.0. The molecular weight of *C. hamatus* SOD1, as determined by gel filtration, was about 33,600 Da. The molecular weight of the monomer, as estimated by SDS-PAGE in the presence of 2-mercaptoethanol, was 16,700 Da. This datum was confirmed by the result of MALDI-TOF mass spectrometry analysis (Figure S3).

The results obtained by atomic absorption analysis showed that one SOD1 molecule contains two copper and two zinc atoms. These data indicated that the isolated protein is a Cu,Zn SOD, as confirmed by the activity that disappeared in the presence of KCN and H_2_O_2_ and by the absence of iron and manganese in the protein solution.

N-terminal sequencing identified a small fragment of the protein, consisting of 22 amino acids: VIKAVCVLKGAGEASGTVFFEQ.

### 3.3. Phylogenetic Relationships and Molecular Modeling

**Figure 1:**
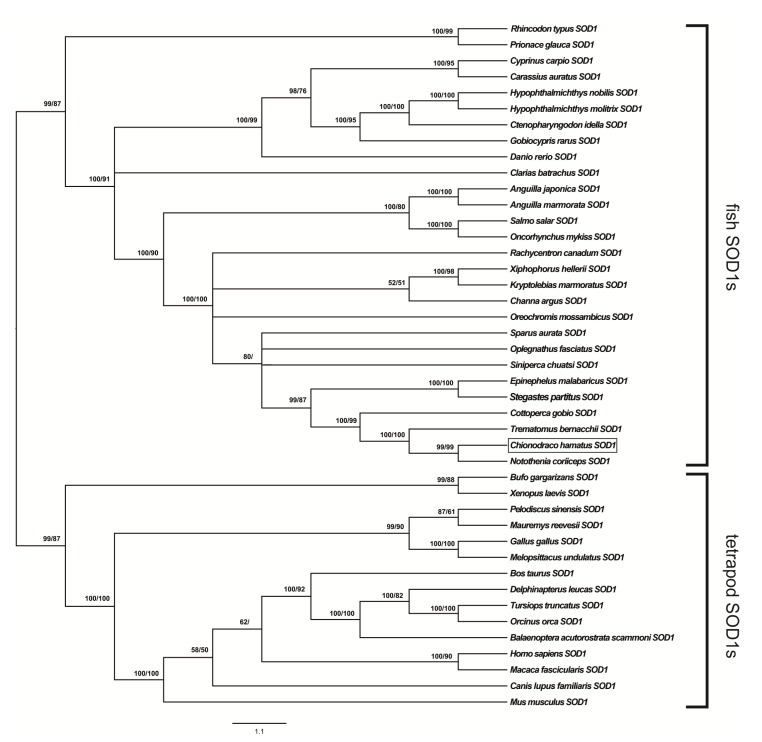
Phylogenetic relationships among SOD1s of various organisms reconstructed on the basis of the cDNA coding region sequences and using both Bayesian interference (BI) and maximum likelihood (ML) methods. Bayesian posterior probability (first number) and bootstrap values higher than 50% are indicated on each node, respectively. The scale for branch length (1.1 substitution/site) is shown below the tree. *C. hamatus* SOD1 is boxed.

The GTR + I + G model was determined to be the best-fit model of SOD1 molecular evolution at the cDNA level. Using all statistical criteria (–lnL = 8591.61), the obtained gamma shape value (four rate categories) was 1.51. BI and ML methods generated phylogenies with the same topology, as illustrated in the cladogram shown in Figure 1.

*C. hamatus* SOD1 was clustered with the fish SOD1s and separated from the orthologs from other vertebrates (posterior probability 99%, bootstrap value 87%; Figure 1). In particular, the three Antarctic SOD1 sequences were grouped together (posterior probability 100%, bootstrap value 100%), a result that was coherent with the abovementioned high similarity of the sequences of *N. coriiceps* and *T. bernacchii*. The sister sequence of Antarctic fish SOD1s was the orthologous protein from the sub-Antarctic notothenioid *C. gobio* (posterior probability 100%, bootstrap value 99%). The cluster including the SOD1s from *Epinephelus malabaricus* and *S. partitus* (Perciformes) was identified as the sister group of notothenioid SOD1s (posterior probability 99%, bootstrap value 87%).

The WAG + I + G model was determined to be the best-fit model of SOD1 molecular evolution at the amino acid level. Using all statistical criteria (–lnL = 3847.83), the obtained gamma shape value (four rate categories) was 0.53. The generated phylogenetic topology, which was the same for both BI and ML methods, is illustrated in the cladogram shown in Figure S4.

The *C. hamatus* SOD1 fell in the fish SOD1 cluster. Desite the lower degree of resolution of this reconstruction, the phylogenetic relationships among the various SOD1 sequences were also confirmed. One difference identified included the close relationships (posterior probability 80%) among the notothenioid fish SOD1s and the orthologous sequences of *Channa argus* (Anabantiformes), *Kryptolebias marmoratus,* and *Xiphophorus hellerii* (Cyprinodontiformes; Figure S4).

An evolution of fish SOD1s characterized by a strong prevalence of negative selection was the result of the MEC analysis. However, during molecular evolution some amino acid residues were subject to significant positive selection (Figure S5). In fact, positive selection was highlighted for Leu^3^, Tyr^20^, Ser^25^, Ser^27^, Ala^78^, Glu^79^, Ala^92^, Asp^99^, Leu^105^, Thr^108^, Pro^110^, and Tyr^111^ (with reference to *Siniperca chuatsi* SOD1 for residue numbering).

The unclock-like hypothesis for teleost SOD1 evolution was confirmed by an equality test of evolutionary rate at both the amino acid (*p* < 0.05) and nucleotide (*p* < 0.0001) level.

**Figure 2:**
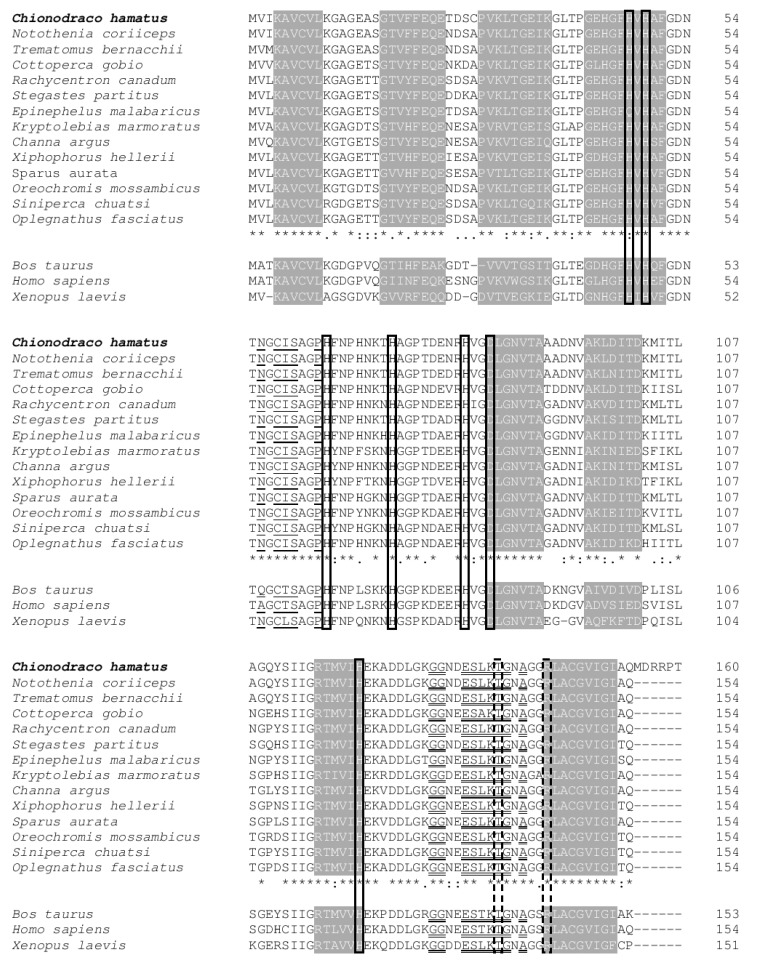
Multiple alignment of amino acid sequences of 14 SOD1s from teleost species, as well as from *Xenopus laevis*, *Bos Taurus,* and *Homo sapiens*. The shaded letters refer to the β-strand conformation of the bovine structure. Unshaded letters between β-strand regions refer to the 7 loops. The boxed letters refer to the amino acids that play an important role in the Cu^2+^–Zn^2+^ coordination environment (solid line) and in the active site region (dotted line). Underlined and double-underlined letters refer to the amino acids that play important roles in the formation of the lower and upper rims, respectively. The symbols at the bottom of the teleost sequences correspond to the definitions in the MUSCLE program: (*) fully conserved; (:) highly conserved; (.) conserved substitution.

High conservation of amino acids that play an important role for dismutase activity, in particular those that are essential for Cu^2+^–Zn^2+^ coordination and in active site region building, was highlighted by multiple sequence alignment among the deduced amino acid sequences of SOD1 from *C. hamatus*, other fish species, and some tetrapods (Figure 2). The β-strand and loop conformations, as well as the amino acids involved in the formation of the lower and upper rims in the teleost proteins, were putatively deduced from the bovine structure. High conservation levels were also evident in teleost SOD1 motifs.

The electrostatic surfaces of SOD1 models displayed a strongly negative shape, as shown in Figure S6. *T. bernacchii* SOD1 (panel A) presented the most negative charge compared to the other analyzed proteins. Conversely, *S. partitus* SOD1 (panel B) presented the least negative charge. The proteins of *N. coriiceps* and *C. hamatus* (panels C and D, respectively) were considerably less negatively charged than *T. bernacchii* SOD1, but more negatively charged than *S. partitus*.

### 3.4. Gene Expression

**Figure 3:**
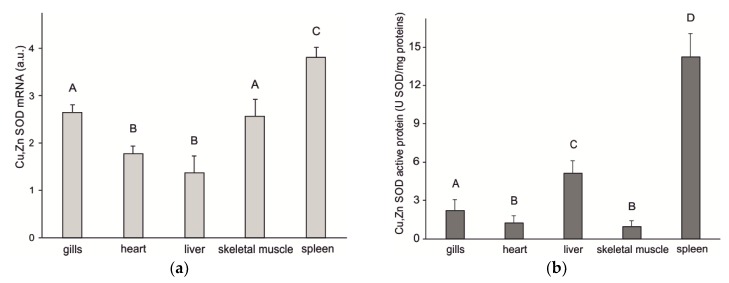
Levels in gills, heart, liver, spleen, and skeletal muscle of *C. hamatus*. (**a**) mRNA; (**b**) active protein. Means with different letters (A–D) are significantly different at *p* < 0.05 (Student–Newman–Keuls *t*-test).

mRNA expression levels were analyzed in the gills, heart, liver, spleen, and skeletal muscle of nonstressed specimens of *C. hamatus* to evaluate the basal tissue specific expression of SOD1. Figure 3a shows that the gene was highly expressed in spleen. This tissue displayed a SOD mRNA expression approximately 1.5-fold higher than gills and skeletal muscle, and 2.5-fold higher than heart and liver tissues (*p* < 0.05).

The expression of active protein determined in these organs again showed a clear prevalence in spleen (Figure 3b). Among the other considered organs, liver tissue showed the highest level of expression, approximately 2.5-fold higher than gills, 4-fold higher than heart, and 5.5-fold higher than skeletal muscle (*p* < 0.05).

## 4. Discussion

Virtually all eukaryotic cells contain Cu,Zn SODs [37,38,39,40,41,42], as well as other anti-stress proteins that allow species to withstand environmental stresses of both natural and anthropogenic origin [43,44,45,46,47,48,49,50,51,52,53]. Our results indicated that a gene coding for cytosolic Cu,Zn SOD is present in the genome of the Antarctic icefish *C. hamatus* and is expressed as active protein in various organs. In particular, the results obtained by the characterization of the purified SOD1 protein confirmed that the *C. hamatus* enzyme maintains the molecular properties of this family of proteins, including the quaternary structure of homodimer, even if the molecular weight of each monomer is unconventional (16,670.37) due to the presence of six extranumerary amino acids at the C-terminal. pI values of five were characteristic of most Cu,Zn SODs, although it is well known that there are some proteins with slightly alkaline or close to neutral pH [7].

Teleost SOD1s can be assumed to have the same or highly similar three-dimensional structures as human, bovine, or frog because the amino acids of structural and functional significance have very similar distribution in the protein primary sequence. The variability of amino acids at each site increases as a function of the distance from the active site. For example, the sequence loop IV (positions 52 to 83), which contribute to the formation of the active site, is highly conserved (72% identity, 88% similarity). The teleost SOD1s present only few amino acid variants in this domain, which may be considered characteristic of the class in comparison with those of other vertebrates.

Even in fish SOD1, the portions necessary for the stability of active site geometry, the β-barrel structure, and for formation of the SOD dimer interface exhibit a high degree of conservation. In particular, both the sub-loop in position 52–64, which is known to protrude from the protein core involved in the unique disulfide bridge, and the small β-sheet at positions 84–90, which supports the catalytic center, are fully conserved.

All the amino acids involved in coordination of the copper (His^47^, His^49^, His^64^, and His^121^) and zinc (His^64^, His^72^, His^81^, and Asp^84^) ions are fully conserved in *C. hamatus* and other fish. The bridging ligand His^64^ delineates a plane that approximately contains both metal ions, as observed in bovine and yeast SOD1s [54,55].

The amino acids that form the lower (positions 56, 58–60, 63) and upper (130–131, 134–139, 141) rims of the electrostatic channel, which define the entrance to the active site, are conserved in teleosts with very few exceptions. Leu^136^ is also important for the overall shape and strength of the electrostatic field around the active site belonging to the second shell, together with residues located at positions 122–123 and 132 [6].

Two other residues, namely Thr^138^, which narrows the active site channel, and Arg^144^, which guides the superoxide anion toward the catalytic Cu^2+^ coordination site via its positively charged guanidinium group, are rigorously conserved.

Teleost SOD1s present additional 11 amino acids which are relevant for the overall structure and function of SOD (Gly^17^, Leu^39^, Gly^45^, Phe^46^, Gly^62^, Pro^67^, Gly^83^, Leu^107^, Gly^139^, Gly^142^, and Gly^148^); these are conserved without exception in the protein family. Finally, two evolutionary unvarying cysteines (positions 58 and 147) form the single disulphide bridge of the protein, which contributes to the stability of the SOD1 tertiary structure.

Although *C. hamatus* SOD1 shows some differences in various positions of the protein, all these observations highlight that the residues that play a significant role in the catalytic mechanism of this enzyme are fully conserved.

A fundamental characteristic that distinguishes the SOD1 of *C. hamatus* from that of all other teleosts is the presence of six extranumerary amino acids at the C-terminal of the protein. By comparing the SOD1 nucleotide sequences of the Antarctic fish, it is possible to observe that this is the result of a large deletion at codon 154, which involves at least the entire portion of the 3’UTR. The analysis of the genome of a species belonging to the same genus (*Chionodraco myersi*), which was recently published [56], could determine the exact extent of this deletion. It is known that the genome of the icefish progenitor underwent deletions, the best known being the globin genes, which gives these fish the characteristic inability to synthesize hemoglobin, which is unique among vertebrates. The data now available do not allow us to make hypotheses about the possible function of this protein portion, but what we can certainly say is that it does not affect the catalytic activity of the enzyme. It would be interesting to verify whether this characteristic is also present in other species of the Channichthyidae family.

The results obtained with phylogenetic analyses support the idea that the molecular evolution of SOD1 progressed in parallel with the evolution of fish orders and, as expected, the SOD1 of *C. hamatus* derived from the same ancestor of the Perciformes clade. This finding confirmed results from a previous study based on the analysis of SOD1s [12].

The similar, but not identical, topology obtained by analyzing SOD1 amino acid sequences suggested that several nucleotide substitutions, although limited, are nonsynonymous, producing higher variability at the amino acid level. This result indicated that purifying selection may have had a significant impact on the evolution of SOD1, a hypothesis supported by the results of MEC analyses. The action of purifying selection on the evolution of the SOD1 family guaranteed the maintenance of its function. However, it is also true that positive selection is one of the primary sources of evolutionary innovation and drives species adaptation in new environments [57]. In fact, many protein families, including those involved in immunity, reproduction, and cell signaling, were subjected to significant positive selection during their evolution [58,59,60].

Despite a strong prevalence of negatively selected amino acids, some residues of fish SOD1 could be under strong positive selection, potentially reflecting peculiar physiological adaptations. One specific physiological feature of *C. hamatus* and other Antarctic fish is thermal adaptation. Antarctic fish evolved in an environment characterized by a very low and constant temperature that increases gas solubility (dioxygen in particular), resulting in a condition where high oxygen concentration in body fluids increases the rate of formation of reactive oxygen species and oxidative stress risk [61,62]. Because these animals remained isolated in Antarctic waters for 20–22 million years exposed to this selective pressure, such conditions may have affected their metabolic adaptive strategies [63]. In fact, some peculiar morphological, metabolic, and molecular adaptations were reported in these endemic fish, including the utilization of lipids as a primary energy source and increased mitochondrial density [64,65,66,67,68,69], and possibly an efficient antioxidant system [70,71,72].

From this viewpoint, it is interesting that SOD1s of notothenioid fish have a molecular structure characterized by some portions with a highly negative surface charge, which is particularly abundant in the *T. bernacchii* enzyme. This electrostatic surface is present in other antioxidant enzymes from Antarctic fish, such as peroxiredoxins [73,74], and could constitute a possible molecular adaptation to low temperatures. As a partial confirmation of this hypothesis, the SOD1 from the tropical perciform *S. partitus* has a lower total negative charge.

Although it is not possible to identify specific amino acids that could be precisely related to molecular cold adaptation in SOD1, some amino acid substitutions exclusive to Antarctic species with respect to more phylogenetically closed non-Antarctic ones could confer a higher degree of flexibility at cold temperatures in notothenioid enzymes [75]. Among these, Ala^15^ replaced Thr, Phe^20^ replaced His or Tyr, Ala^91^ replaced Thr, and Ala^108^ replaced Thr or Asn. From the data now available, it is not possible to discuss without risk whether this divergence is correlated with specific behavioral and physiological features of different species and families, but it is evident that all these amino acid substitutions may be an adaptation to low temperatures [76,77].

The phylogenetic analyses show that Antarctic fish SOD1s constantly emerge as a distinct cluster, an expected result given the high similarity and identity of these sequences. Based on the isolation experienced by Antarctic species in recent geological times, our results indicated that these proteins experienced relatively rapid evolution, which is concurrent with the theory of the unclock-like evolution of Cu,Zn SOD by confirming the erratic differentiation of these proteins [78]. In fact, the literature indicated that Cu,Zn SODs of various organisms showed a rather constant rate of amino acid substitution (approximately 15 aa/100 aa/100 million years) throughout the last 60 million years [79,80,81]. The analysis of SOD1 primary sequences from Antarctic fish confirmed these data with the percentage of amino acid substitution among Antarctic species being 3.2%, which, considering a separation time of 21 million years, corresponds to 15.24 aa/100 aa/100 million years.

As for the analysis of gene expression, the data obtained demonstrated that *C. hamatus* SOD1 was expressed in all analyzed tissues. In particular, the spleen was the organ in which the levels of mRNA and active protein accumulation were the highest. The Antarctic marine environment is rich in microorganisms and parasitic organisms, especially in vertebrates. Parasitism is a very frequent occurrence in Antarctic teleosts, especially by nematodes [82]. The fish spleen is an organ primarily involved in immune defenses, being the site of leukocyte production [83], and ROS can form in large quantities during inflammatory processes and phagocytic cell activity. Therefore, it is important that antioxidant enzymes, such as SOD1, which are responsible for eliminating excessive levels of these molecules, act in a coordinated way with the immune defenses, so as to prevent negative effects of high levels of ROS from extending to host cells.

**Figure 4:**
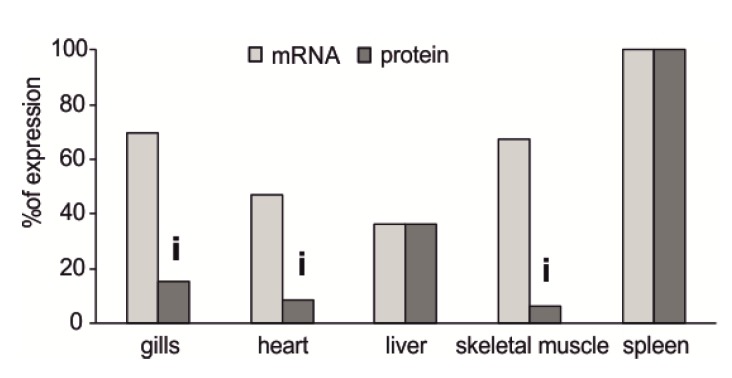
Comparison between the relative expressions of SOD1 mRNA and active protein in the gills, heart, liver, spleen, and skeletal muscle of *C. hamatus*. The symbol “i” above the histogram columns indicates the possible inhibition of translation.

When comparing the percentages of mRNA and protein expression, the levels of active protein in the gills, heart, and skeletal muscle were relatively lower than the messenger levels, suggesting that part of the transcript is not immediately translated (Figure 4). Cytoplasmic foci are known to exist, such as P-bodies or stress granules (SGs), in which these messengers can be stored, undergoing degradation or future translation, respectively [84,85]. This condition is a common feature in organisms that live in stressogenic conditions, but without acute stress [40,86], allowing an extremely rapid response by the tissues toward the sudden onset of acute stress, specifically, a greater biosynthesis of SOD1 in response to increased ROS formation rate. This condition easily occurs in the gills, heart, and skeletal muscle of *C. hamatus* in relation to its peculiar absence of hemoglobin, which entails chronic hypoxia for the animal and which becomes even more extreme when engaged in locomotor activity, which requires increased oxygen consumption of skeletal muscle and the cardiocirculatory and respiratory systems.

Nucleation proteins are involved in SG formation, such as the T-cell-restricted intracellular antigen (TIA) proteins, TIA-1 and TIA-1-related protein (TIAR), which both self-associate to promote the growth of SGs, directly binding target RNAs. Recently, we characterized some variants of TIA-1 in *C. hamatus* and *T. bernacchii;* preliminary data indicated that, in both species, high levels of expression of the messenger of TIA-1 correspond to low levels of biosynthesis of antioxidant enzymes, such as peroxiredoxins, supporting the hypothesis of post-transcriptional control operated by stress granules [87,88,89].

## 5. Conclusions

The results obtained in this work showed that *C. hamatus*, as well as the other Antarctic teleost species studied so far, is able to cope with the risk of oxidative stress thanks to an antioxidant defense system in which SOD1 constitutes an important enzyme component. Therefore, the data presented here constitute a further important contribution to the knowledge of the physiological antioxidant defenses of these animals, which evolved under selective pressure represented by the constant production of high levels of ROS. The small number of characterized antioxidant enzymes from Antarctic fish represents a limitation of phylogenetic and physiological analyses; the implementation of this research could provide a more realistic picture of the evolution of these anti-stress proteins and may help to predict what impact temperature changes and other environmental perturbations associated with global climate change could have on the ichthyofauna of the frozen continent.

## Supplementary Materials

The following are available online at https://www.mdpi.com/2076-3921/9/4/325/s1. Figure S1: Chromatographic analysis of Cu,Zn SOD by ion exchange FPLC; Figure S2: cDNA sequence of *C. hamatus* SOD1 and deduced amino acid sequence; Figure S3: MALDI-TOF spectrum of *C. hamatus* SOD1; Figure S4: Phylogenetic relationships among SOD1s of various organisms, reconstructed on the basis of amino acid sequences and using both BI and ML methods; Figure S5: Detection of selective pressure on SOD1 using mechanistic empirical combination (MEC); Figure S6: Potential isosurfaces are shown at +3 kT/e in blue and –3 kT/e in red for SOD1 of *T. bernacchii*, *S. partius*, *N. coriiceps,* and *C. hamatus*; Table S1: Sequences and melting temperatures of primers used for PCR amplification of *C. hamatus SOD1*; Table S2: Amino acid sequences of SOD1 (and their accession numbers in GenBank) used for phylogenetic reconstruction; Table S3: Summary of the investigated proteins and the relative selected templates for structural model building; Table S4: Percentage of identity calculated by comparing SOD1 amino acid sequence of the *C. hamatus* with those of other vertebrates; Table S5: Purification of SOD1 from *C. hamatus* livers.
