# Supplementary material for: Copper/Zinc Superoxide Dismutase from the Crocodile Icefish Chionodraco hamatus: Antioxidant Defense at Constant Sub-Zero Temperature
Source: Antioxidants (Basel). 2020 Apr 17;9(4):325. doi: 10.3390/antiox9040325 (PMC7222407; doi:10.3390/antiox9040325)
Supplement: Supplementary file 1 [file antioxidants-09-00325-s001.zip › Table S2.docx]

**Table S2:** Amino acid sequences of SOD1 (and their accession numbers in GenBank) used for phylogenetic reconstruction.

| **SPECIES** | **ACCESSION NUMBER** |
| --- | --- |
| *Anguilla japonica* | AB558958.1 |
| *Anguilla marmorata* | KR350468.1 |
| *Balaenoptera acutorostrata scammoni* | XM_007183692.2 |
| *Bos taurus* | NM_174615.2 |
| *Bufo gargarizans* | DQ437103.1 |
| *Canis lupus familiaris* | NM_001003035.1 |
| *Carassius auratus* | JX977106.1 |
| *Channa argus* | MF078485.1 |
| *Chionodraco hamatus* | AY736281.1 |
| *Clarias batrachus* | KF444052.2 |
| *Cottoperca gobio* | XM_029448511.1 |
| *Ctenopharyngodon idella* | GU901214.1 |
| *Cyprinus carpio* | JF342355.1 |
| *Danio rerio* | NM_131294.1 |
| *Delphinapterus leucas* | XM_022568431.1 |
| *Epinephelus malabaricus* | AY035854.1 |
| *Gallus gallus* | U28407.1 |
| *Gobiocypris rarus* | KF515699.1 |
| *Homo sapiens* | AY450286.1 |
| *Hypophthalmichthys molitrix* | HM469964.1 |
| *Hypophthalmichthys nobilis* | HM469965.1 |
| *Kryptolebias marmoratus* | EU116027.1 |
| *Macaca fascicularis* | NM_001285406.1 |
| *Mauremys reevesii* | JX843790.1 |
| *Melopsittacus undulatus* | AY241393.1 |
| *Mus musculus* | NM_011434.2 |
| *Notothenia coriiceps* | XM_010772065.1 |
| *Oncorhynchus mykiss* | AF469663.1 |
| *Oplegnathus fasciatus* | AY613390.1 |
| *Orcinus orca* | XM_004264510.2 |
| *Oreochromis mossambicus* | AY491056.1 |
| *Pelodiscus sinensis* | JX470524.1 |
| *Prionace glauca* | P11418.1 |
| *Rachycentron canadum* | DQ907939.1 |
| *Rhincodon typus* | XM_020511155.1 |
| *Salmo salar* | AY736282.1 |
| *Siniperca chuatsi* | KJ558392.1 |
| *Sparus aurata* | JQ308832.1 |
| *Stegastes partitus* | XM_008277879.1 |
| *Trematomus bernacchii* | AY736280.1 |
| *Tursiops truncatus* | XM_004322979.2 |
| *Xenopus laevis* | BC070696.1 |
| *Xiphophorus hellerii* | HM241653.1 |
