# Supplementary material for: Copper/Zinc Superoxide Dismutase from the Crocodile Icefish Chionodraco hamatus: Antioxidant Defense at Constant Sub-Zero Temperature
Source: Antioxidants (Basel). 2020 Apr 17;9(4):325. doi: 10.3390/antiox9040325 (PMC7222407; doi:10.3390/antiox9040325)
Supplement: Supplementary file 1 [file antioxidants-09-00325-s001.zip › Table S3.docx]

**Table S3.** Summary of the investigated proteins and the relative selected templates for structural model building. Species, template PDB ID, sequence identity (%), oligomeric state and QMEAN values are provided (see text for details).

| species | template PDB ID | sequence identity (%) | oligo-state | QMEAN |
| --- | --- | --- | --- | --- |
| *Chionodraco hamatus* | 5yto | 68.83 | homo-dimer | 1.64 |
| *Cottoperca gobio* | 5yto | 71.43 | homo-dimer | 2.07 |
| *Notothenia coriiceps* | 5yto | 68.83 | homo-dimer | 1.54 |
| *Rachycentron canadum* | 5yto | 70.13 | homo-dimer | 1.36 |
| *Stegastes partitus* | 5yto | 68.83 | homo-dimer | 1.55 |
| *Trematomus bernacchii* | 5yto | 68.83 | homo-dimer | 1.53 |
