# Supplementary material for: Copper/Zinc Superoxide Dismutase from the Crocodile Icefish Chionodraco hamatus: Antioxidant Defense at Constant Sub-Zero Temperature
Source: Antioxidants (Basel). 2020 Apr 17;9(4):325. doi: 10.3390/antiox9040325 (PMC7222407; doi:10.3390/antiox9040325)
Supplement: Supplementary file 1 [file antioxidants-09-00325-s001.zip › Table S4.docx]

**Table S4:** Percentage of identity calculated by comparing SOD1 amino acid sequences of the *C. hamatus* with those of other vertebrates.

| **species** | **% identity** |
| --- | --- |
| *Notothenia coriiceps* | 98.70% |
| *Trematomus bernacchii* | 96.03% |
| *Rachycentron canadum* | 88.96% |
| *Stegastes partitus* | 89.61% |
| *Epinephelus malabaricus* | 88.96% |
| *Cottoperca gobio* | 87.66% |
| *Oplegnathus fasciatus* | 87.01% |
| *Sparus aurata* | 85.06% |
| *Siniperca chuatsi* | 85.06% |
| *Oreochromis mossambicus* | 84.42% |
| *Channa argus* | 84.42% |
| *Oncorhynchus mykiss* | 82.47% |
| *Anguilla japonica]* | 82.47% |
| *Salmo salar* | 81.82% |
| *Anguilla marmorata* | 81.82% |
| *Xiphophorus hellerii* | 81.17% |
| *Cyprinus carpio* | 81.17% |
| *Danio rerio* | 80.52% |

%
