# Supplementary material for: Copper/Zinc Superoxide Dismutase from the Crocodile Icefish Chionodraco hamatus: Antioxidant Defense at Constant Sub-Zero Temperature
Source: Antioxidants (Basel). 2020 Apr 17;9(4):325. doi: 10.3390/antiox9040325 (PMC7222407; doi:10.3390/antiox9040325)
Supplement: Supplementary file 1 [file antioxidants-09-00325-s001.zip › Table S5.docx]

**Table S5.** Purification of SOD1 from *C. hamatus* liver.

| **Fraction** | **Volume (ml)** | **Total protein (mg/ml)** | **Total activity (units/ml)** | **Specific activity (units/mg)** | **Purification factor** |
| --- | --- | --- | --- | --- | --- |
|  |  |  |  |  |  |
|  |  |  |  |  |  |
| Crude extract | 98.6 | 22.83 | 171.92 | 7.53 | 1 |
| Sephadex G-75 | 61.9 | 1.09 | 73.01 | 66.98 | 9 |
| DEAE A-50 | 29.8 | 0.74 | 81.07 | 109.55 | 15 |
| Mono Q | 7.6 | 0.21 | 141.83 | 675.38 | 90 |
