# Supplementary material for: Copper/Zinc Superoxide Dismutase from the Crocodile Icefish Chionodraco hamatus: Antioxidant Defense at Constant Sub-Zero Temperature
Source: Antioxidants (Basel). 2020 Apr 17;9(4):325. doi: 10.3390/antiox9040325 (PMC7222407; doi:10.3390/antiox9040325)
Supplement: Supplementary file 1 [file antioxidants-09-00325-s001.zip › Figure S2.docx]

**Figure S2.** cDNA sequence of *C. hamatus* SOD1 and deduced amino acid sequence. Bold type: nucleotides of coding region. Derived amino acid sequence given below. 5’ and 3’ UTR regions are underlined. Putative polyadenylation (++++) signal is indicated.

1 actataacagtt

13 ggttgtctttagctagcaggagcaatagcaacttggcgtcctttgaaactactgcaaac

72 **atg gtt ata aaa gcg gtg tgt gta ttg aaa gga gct gga gag gct**

*Met Val Ile Lys Ala Val Cys Val Leu Lys Gly Ala Gly Glu Ala*

117 **agc ggg act gtc ttc ttc gag cag gag act gat tca tgc cct gtg**

*Ser Gly Thr Val Phe Phe Glu Gln Glu Thr Asp Ser Cys Pro Val*

162 **aag ctg acc gga gaa atc aaa ggc ctt act cct ggt gag cat ggt**

*Lys Leu Thr Gly Glu Ile Lys Gly Leu Thr Pro Gly Glu His Gly*

207 **ttc cat gtc cat gct ttt gga gac aat aca aac ggg tgc atc agt**

*Phe His Val His Ala Phe Gly Asp Asn Thr Asn Gly Cys Ile Ser*

252 **gca ggc cct cac ttc aat ccc cac aac aag act cat gcc ggt cct**

*Ala Gly Pro His Phe Asn Pro His Asn Lys Thr His Ala Gly Pro*

297 **act gat gaa aat agg cat gtt gga gac ctg ggg aat gtg act gct**

Thr Asp Glu Asn Arg His Val Gly Asp Leu Gly Asn Val Thr Ala

342 **gca gct gat aat gtt gca aag ctc gac atc acg gac aag atg atc**

Ala Ala Asp Asn Val Ala Lys Leu Asp Ile Thr Asp Lys Met Ile

387 **acc ctt gct ggc caa tac tct att att ggc aga acc atg gtg att**

Thr leu Ala Gly Gln Tyr Ser Ile Ile Gly Arg Thr Met Val Ile

432 **cat gag aag gcc gac gac ctg gga aaa gga ggc aat gat gag agt**

His Glu Lys Ala Asp Asp Leu Gly Lys Gly Gly Asn Asp Glu Ser

477 **cta aag aca ggc aat gct ggt gga cgt ctg gcc tgt gga gtc atc**

Leu Lys Thr Gly Asn Ala Gly Gly Arg Leu Ala Cys Gly Val Ile

522 **ggc atc gcc caa atg gac agg aga ccg aca tga** ctatcaaaggactag

Gly Ile Ala Gln Met Asp Arg Arg Pro Thr

570 cagcagtagcagcaggactagcagcatataatgatgttaaatgtgttttaggaacatcc

629 attgcaaatacatggaattcaataaacattgaatagcacg

++++++
