# Supplementary material for: Copper/Zinc Superoxide Dismutase from the Crocodile Icefish Chionodraco hamatus: Antioxidant Defense at Constant Sub-Zero Temperature
Source: Antioxidants (Basel). 2020 Apr 17;9(4):325. doi: 10.3390/antiox9040325 (PMC7222407; doi:10.3390/antiox9040325)

**Figure S4.** Phylogenetic relationships among SOD1s of various organisms, reconstructed on the basis of amino acid sequences and using both BI and ML methods. Bayesian posterior probability (first number) and bootstrap values higher than 50% are indicated on each node, respectively. The scale for branch length (1.1 substitution/site) is shown below the tree. *C. hamatus* SOD1 is boxed.


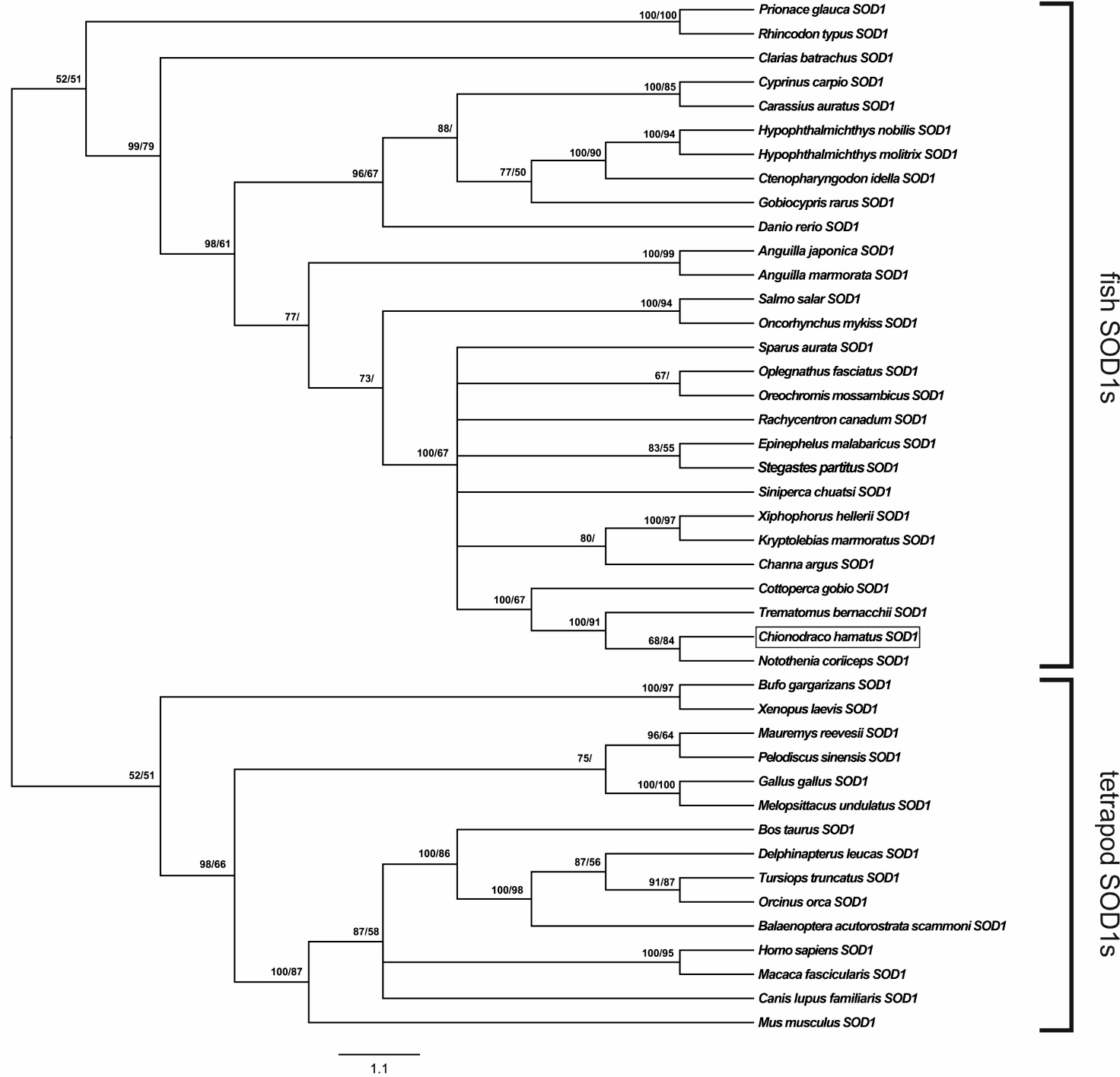

Supplement: Supplementary file 1 [file antioxidants-09-00325-s001.zip › Figure S4.docx]
