# Supplementary material for: Copper/Zinc Superoxide Dismutase from the Crocodile Icefish Chionodraco hamatus: Antioxidant Defense at Constant Sub-Zero Temperature
Source: Antioxidants (Basel). 2020 Apr 17;9(4):325. doi: 10.3390/antiox9040325 (PMC7222407; doi:10.3390/antiox9040325)
Supplement: Supplementary file 1 [file antioxidants-09-00325-s001.zip › Figure S5.docx]

**Figure S5:** Detection of selective pressure on SOD1 using the mechanistic empirical combination (MEC) model based on human protein tertiary structure (pdb: 5YTO) and mapped on *Siniperca chuatsi* SOD1, where the yellow areas indicate the positively selected sites. The color scale illustrates the intensity of selective constraints.

1 11 21 31

M V L K A V C V L R G D G E T S G T V Y F E Q E S D S A P V K L T G Q I K G L T

41 51 61 71

P G E H G F H V H V F G D N T N G C I S A G P H Y N P H G K N H A G P N D A E R

81 91 101 111

H V G D L G N V T A G A D N V A K I D I T D K M L S L T G P Y S I I G R T M V I

121 131 141 151

H E K A D D L G K G G N E E S L K T G N A G G R L A C G V I G I T Q

Legend:

The selection scale:

| 1   2   3   4   5   6   7 |
| --- |
| Positive selection Purifying selection |
