# Supplementary material for: Copper/Zinc Superoxide Dismutase from the Crocodile Icefish Chionodraco hamatus: Antioxidant Defense at Constant Sub-Zero Temperature
Source: Antioxidants (Basel). 2020 Apr 17;9(4):325. doi: 10.3390/antiox9040325 (PMC7222407; doi:10.3390/antiox9040325)

**Figure S6.** Potential isosurfaces are shown at +3 kT/e in blue and -3 kT/e in red for SOD1 of *T. bernacchii*, *S. partius*, *N. coriiceps* and *C. hamatus* (panels A, B, C and D, respectively).


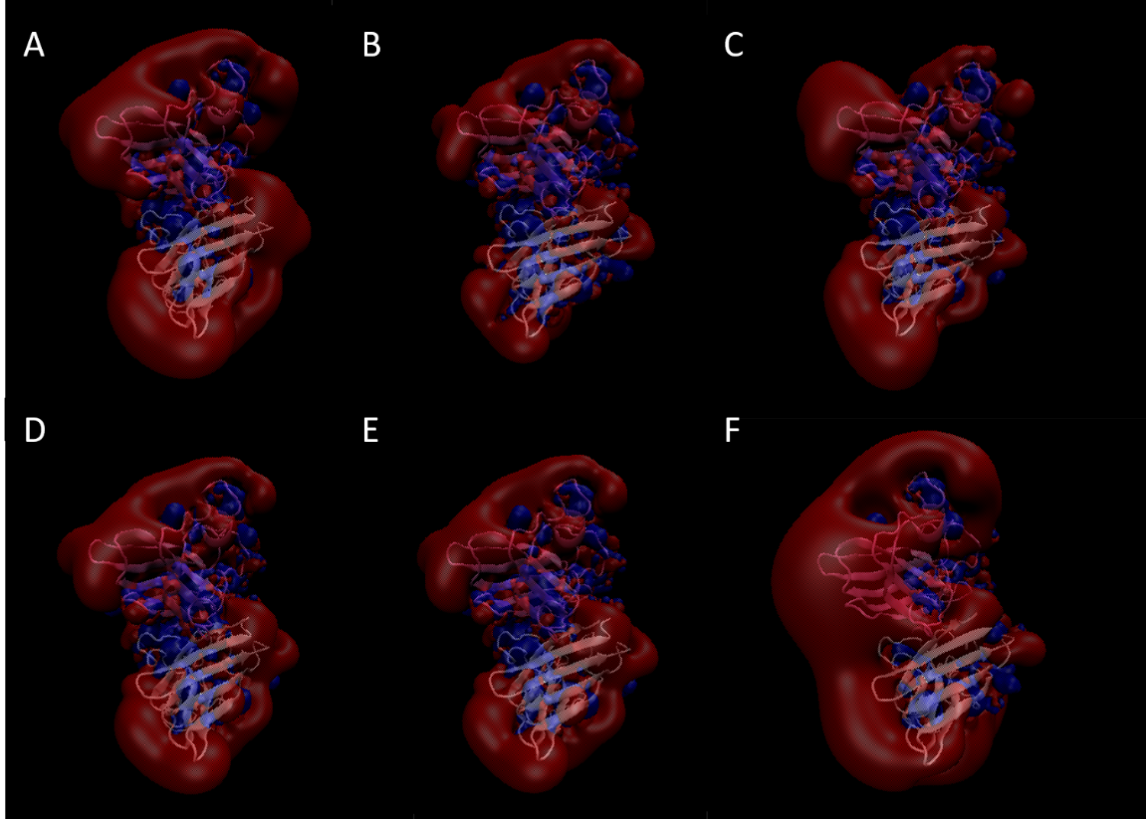

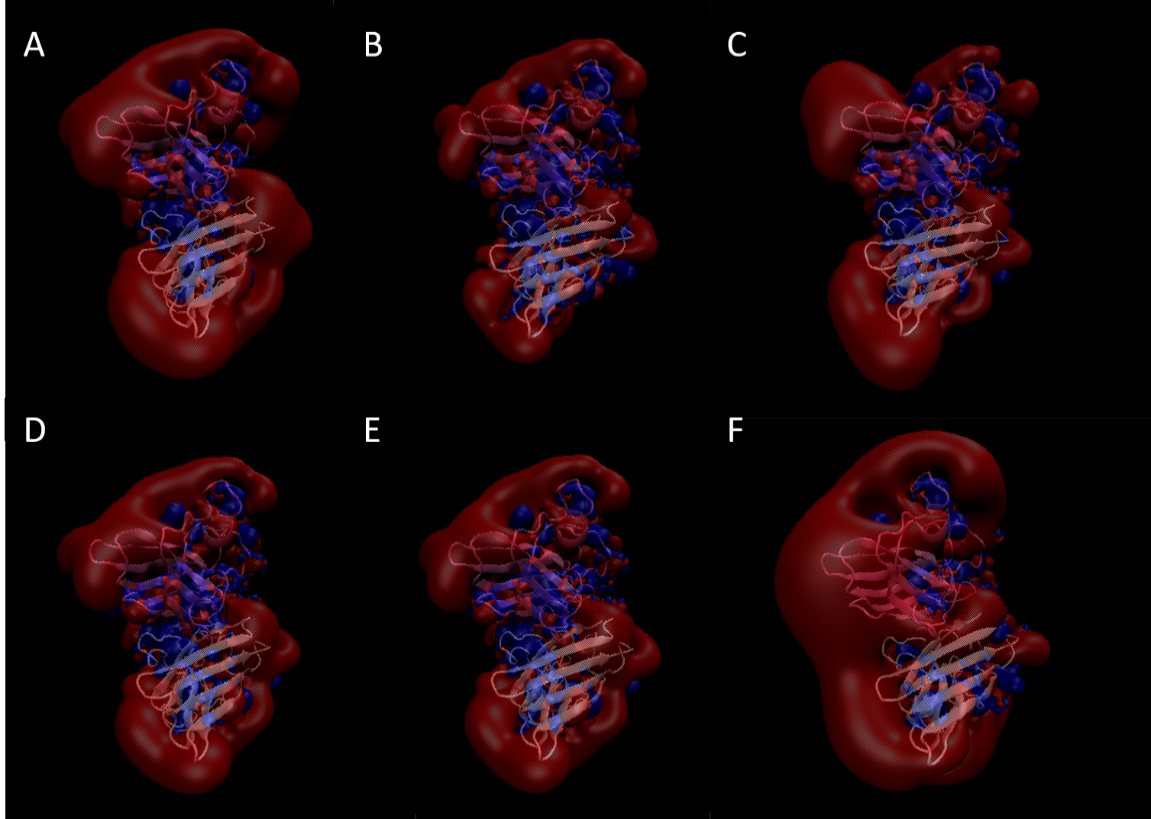

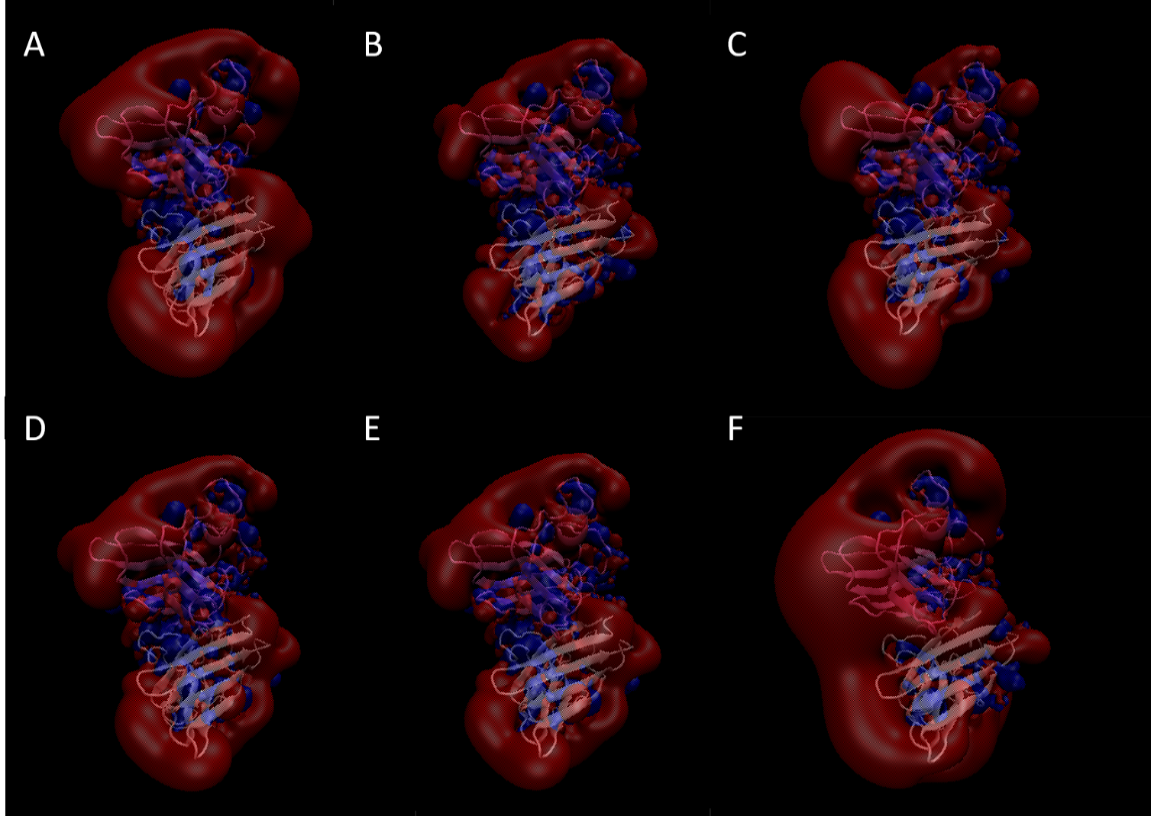

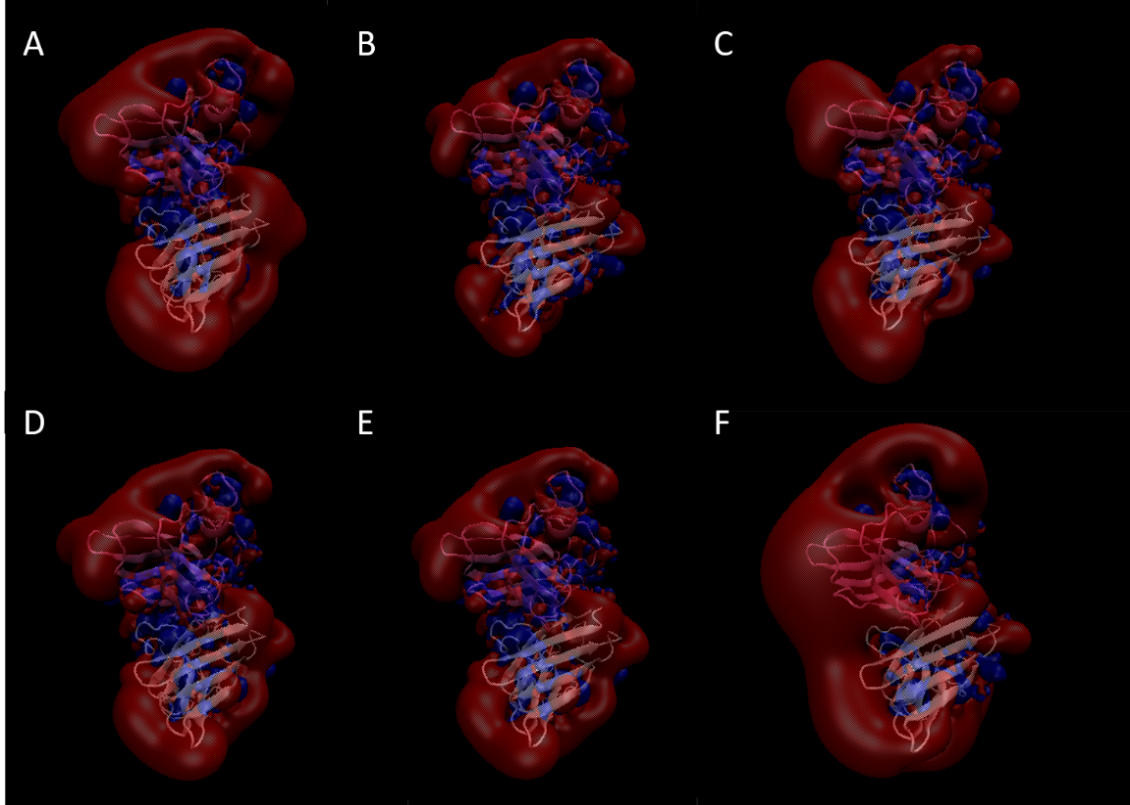

Supplement: Supplementary file 1 [file antioxidants-09-00325-s001.zip › Figure S6.docx]
