# Supplementary material for: Copper/Zinc Superoxide Dismutase from the Crocodile Icefish Chionodraco hamatus: Antioxidant Defense at Constant Sub-Zero Temperature
Source: Antioxidants (Basel). 2020 Apr 17;9(4):325. doi: 10.3390/antiox9040325 (PMC7222407; doi:10.3390/antiox9040325)
Supplement: Supplementary file 1 [file antioxidants-09-00325-s001.zip › Table S1.docx]

**Table S1**: Sequences and melting temperatures of primers used for PCR amplification of *C. hamatus* *sod1*.

| **Primers** | **Sequence 5' - 3'** | **T_m_ (°C)** |
| --- | --- | --- |
| SOD1_Fw1 | AAAGCTGTCTGTGTGTTGAAAGG | 66 |
| SOD1_Fw2 | GACAATACAAACGGGTGCATC | 64 |
| SOD1_Fw4 | ATAGGCATGTTGGAGACCTG | 64 |
| SOD1_Re1 | GAGCTTTGCAACATTATCAG | 59 |
| SOD1_Re2 | ACCTGGGGAATGTGACTGCTG | 70 |
| 3’ RACE Fw1 | AGCGGGACTGTCTTCTTCG | 67 |
| 3’ RACE Fw2 | AAACGGGTGCATCAGTGC | 66 |
| 5’ RACE Re1 | AATAGAGTATTGGCCAGCAA | 61 |
| 5’ RACE Re2 | TTGTTGTGGGGATTGAA | 58 |
| βAct_Fw | GGATGGGACAAGGTTAAGCA | 65 |
| βAct_Re | GAGCCTCCGATCCAGACA | 67 |
| oligo-dT adaptor | ACCACGCGTATCGATGTCG(T)16 | 69 |
| anchor | ACCACGCGTATCGATGTCG | 68 |
| Abridged anchor | GGCCACGCGTCGACTAGTACGGGIIGGGIIGGGIIG | 92 |
| AUAP | GGCCACGCGTCGACTAGTAC | 71 |
| T7 | TAATACGACTCACTATAGGG | 57 |
| SP6 | GATTTAGGTGACACTATAG | 53 |
