# Supplementary figures and images for: Copper/Zinc Superoxide Dismutase from the Crocodile Icefish Chionodraco hamatus: Antioxidant Defense at Constant Sub-Zero Temperature
Source: Antioxidants (Basel). 2020 Apr 17;9(4):325. doi: 10.3390/antiox9040325 (PMC7222407; doi:10.3390/antiox9040325)

**Figure S3.** MALDI-TOF spectrum of *C. hamatus* SOD1


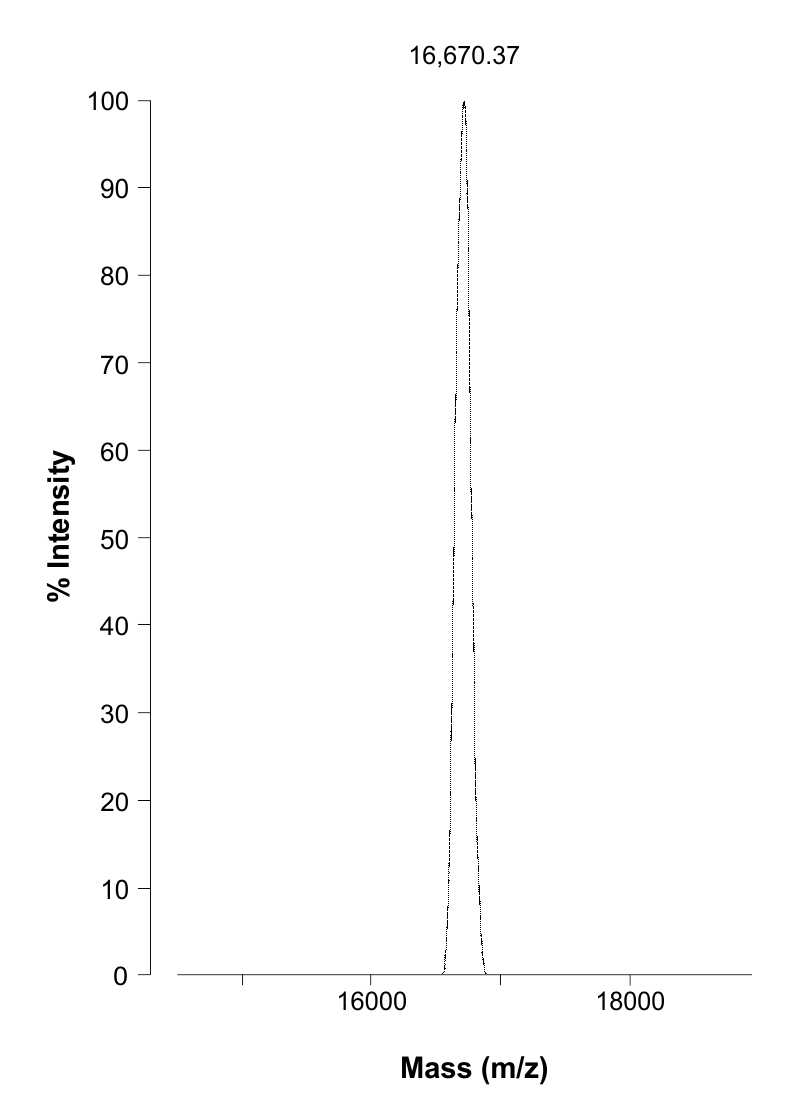

Supplement: Supplementary file 1 [file antioxidants-09-00325-s001.zip › Figure S3.docx]
